# Tolerant, Growing Cells from Nutrient Shifts Are Not Persister Cells

**DOI:** 10.1128/mBio.00354-17

**Published:** 2017-04-18

**Authors:** Jun-Seob Kim, Thomas K. Wood

**Affiliations:** Department of Chemical Engineering, Pennsylvania State University, University Park, Pennsylvania, USA; New York University

**Keywords:** antimicrobial agents, persistence, tolerance

## Abstract

There is much controversy about the metabolic state of cells that are tolerant to antibiotics, known as persister cells. In this opinion piece, we offer an explanation for the discrepancy seen: some laboratories are studying metabolically active and growing cell populations (e.g., as a result of nutrient shifts) and attributing the phenotypes that they discern to persister cells while other labs are studying dormant cells. We argue here that the metabolically active cell population should more accurately be considered tolerant cells, while the dormant cells are the true persister population.

## OPINION/HYPOTHESIS

**FIG 1:**
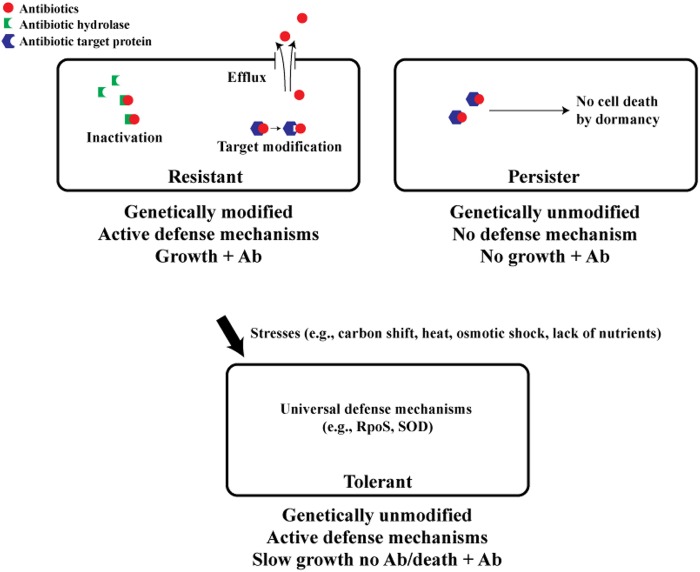
Major mechanisms used by bacteria to survive antibiotics. Resistance is the use of the active defense mechanism of mutation to withstand antibiotic (Ab) stress; surviving cells grow in the presence of the antibiotic, and offspring inherit the phenotype. The mutations include those that inactivate antibiotics by increasing efflux, by target modification, and by direct antibiotic modification. Persistence is the cessation of cellular activity (i.e., dormancy) that allows cells to not grow in the presence of antibiotics but basically to not change in concentration. The persistence phenotype is not inherited, and cells revert rapidly to wild-type growth once the antibiotic stress is removed and nutrients are presented. Tolerance is due to slow growth prior to the antibiotic stress, and the slow-growing cells utilize universal defense mechanisms (e.g., RpoS, superoxide dismutase [SOD], and heat/cold shock proteins) to counter various environmental stresses such as carbon shifts and lack of nutrients. Upon antibiotic addition, the concentration of tolerant cells decreases continually, and the phenotype of tolerance is noninherited.

Persisters are the remaining, genetically unaltered population of bacterial cells that, after an initial die-off, survive prolonged antibiotic treatment with a basically unchanging or slowly decreasing population density due to their lack of metabolic activity (1). In comparison, resistant cells grow in the presence of the antibiotic (as long as adequate carbon sources are available) due to mutations. Tolerant cells grow prior to antibiotic addition and then survive longer than exponentially growing cells in the presence of the antibiotic, but their population usually continues to decrease appreciably (1), and the phenotype is a population-wide phenomenon (2). Given the similarity of these three phenotypes (Fig. 1), there has been a great deal of confusion about the metabolic activity of persister cells (3–5) and the relation of persistence to tolerance. One possible explanation of why some groups claim that persister cells are metabolically active whereas others present evidence that they are dormant is that the way in which one generates these populations matters, i.e., some groups are studying metabolically active and growing tolerant cell populations (and mistakenly calling them persister cells) by using procedures such as nutrient switches to generate their populations of interest. In contrast, others are studying dormant persister cell populations that are obviously nongrowing.

The experimental evidence that indicates that persister cells are nongrowing dates back to the original research that defined and originated the field. Hobby et al. (6) first demonstrated that penicillin is ineffective against metabolically inactive cells by creating nongrowing *Staphylococcus aureus* cells by reducing the culture temperature. Bigger (7) then confirmed these results that persister cells are dormant via three experiments that showed that penicillin is ineffective against persister cells if growth is stopped by reducing the culture temperature, by removing nutrients, or by adding boric acid. Later studies have confirmed this work by demonstrating that persister cells lack transcription, translation, and proton motive force (8) as well as by showing reduced metabolic activity by sorting cells based on weak production of an unstable green fluorescent protein under the control of a ribosomal promoter (9). Studies that claim that persister cells are metabolically active, like that by Wakamoto et al. (10), usually have a major flaw in this context (11); in this case, the cells that survived the prodrug isoniazid due to low activity of the enzyme required to activate the prodrug (catalase) are not proof that persister cells are metabolically active but instead are proof of the noise that is inherent in cellular metabolism. Hence, persister cells are dormant and nongrowing.

Many researchers have used the designations type I and type II persister cells since the Balaban group coined the terms (12). Type I persister cells are dormant and are true persister cells; these type I persisters were reported to have a growth lag of 14 h in fresh medium (12), whereas others found a lag of about 2 h (23). Critically, the type II “persisters” of the Balaban et al. publication (12) had a low growth rate prior to antibiotic addition. Furthermore, these cells had an inherited phenotype after several rounds of ampicillin treatment, and the cells could grow in the presence of ampicillin. Hence, we believe that these type II cells are not persister cells for three reasons: (i) true persister cells have no inherited phenotype, (ii) they do not grow in the presence of antibiotic, and (iii) they do not grow in the absence of antibiotic.

Proposing an alternative method to produce persister cells, the Brynildsen group subjected *Escherichia coli* cells to a nutrient shift (e.g., glucose to fumarate) and found that the cells were moderately more tolerant to the fluoroquinolone ofloxacin (~50-fold increase) (14). Unfortunately, rather than discerning “a mechanistic persister formation pathway” as the authors claimed (14), they instead studied cells growing exponentially (prior to antibiotic addition) as evidenced by the increase in cell density from 10^4^ cells/ml to 10^6^ cells/ml over the course of 8 h after the nutrient switch. Critically, growth on fumarate alone gave results similar to that of the switch from glucose to fumarate (i.e., only 10-fold-fewer cells that were tolerant to the antibiotic), which indicates that the phenomenon studied was simply the increase in antibiotic tolerance seen in a slow-growing population (15). Moreover, the ensuing conclusions regarding persistence and the stringent response via guanosine tetraphosphate (ppGpp) and DNA gyrase activity based on results from diauxic growth are probably not valid for persisters (but may be valid for cells undergoing nutrient stress). Also, since the changes in tolerant cell populations based on diauxic growth were relatively modest (on the order of 10-fold), they are probably not informative for work in the persister field, where cell populations change on the order of 10^5^ cells/ml (3).

This lack of a robust phenotype and the resulting cell growth may explain how Amato et al. (14) mistakenly thought that they were studying persister cells, which by all accounts in the field do not increase in population size. Hence, there may be disagreement in the persister field about the degree of dormancy, but results in which the cell density is increasing prior to antibiotic treatment should not be attributed to persister cells.

Similarly, the lack of a robust phenotype (~20-fold change in antibiotic tolerance) and their work with growing *E. coli* cells also led to the conclusion by the Brynildsen group that cyclic AMP (cAMP) addition increases persistence (14). Based on changes in actual persister cell populations of 235-fold, 19-fold, and 4,200-fold for three independent lines of evidence related to cAMP, we have found that cAMP instead clearly decreases persistence (16).

In a second paper, which relies on the nutrient shift method, the Brynildsen group claimed to study persister cell formation in biofilms (17). Unfortunately, their results show only modest (~13-fold increase) changes in the ability of nutritionally stressed cells to tolerate antibiotics and show that the cells were growing exponentially prior to antibiotic treatment; so, once again, persister cells were not studied, and the results related to specific *E. coli* mutants are not valid for persister cells.

In a third paper, which relies on the effect of diauxic growth, the Brynildsen group studied the effects of two antibiotics, ampicillin and ofloxacin, and linked the “persistence” of *E. coli* to RelA, ClpA, SsrA, and SmpB as well as concluding that there were differences in the mechanisms for the antibiotic tolerance of cells to the two antibiotics (18). As before, the modest phenotypes (~25-fold increase) and the use of growing cells (prior to antibiotic treatment) invalidate their conclusions for persister cells.

In a fourth paper, which uses nontraditional “persister” cells (19), the Brynildsen group studied “persister cells” generated in stationary cultures; they studied these cells by using fluorescence-activated cell sorting (FACS) in which they utilized redox sensor green staining for determining metabolic activity and mCherry dilution for determining cell growth. Their main conclusion was that “persister cells” are derived from cells with high redox activity and that inhibiting respiration reduces “persisters.” As with their diauxic cultures, the problem with using stationary-phase cells is that the cells are still growing (prior to antibiotic addition); hence, they are not persisters. For example, in Fig. 5g of reference 19, after treatment with potassium cyanide and ampicillin, persister cells are not created since the population continues to decrease with time without the biphasic pattern that is typical of persistence; instead, a pattern consistent with tolerance is seen since the viable cell population continues to decrease. Indeed, previous authors utilizing stationary cells recognize this population as tolerant, not as persister cells (13, 20, 21), and transmission electron microscopy has demonstrated that persister cells are phenotypically distinct from stationary-phase cells (22). Hence, stationary-phase cells are not persister cells. As for other problems in the work by Orman and Brynildsen (19), the authors diluted their sorted cells into rich medium prior to the persister assay, which invalidates the persister assay since almost 50% of the persister cells in stationary cultures lose their tolerant phenotype in several minutes (23).

Other groups have also studied actively growing cells and attributed their results to persister cells. The Heinemann group (24) also used the nutrient switch from glucose to fumarate for *E. coli* to “generate large numbers of persisters present in nutrient rich environments.” Using proteomics, they concluded that “persister” cells depend on an active RpoS system and that “persisters” are metabolically active. Clearly, the cell population was tolerant to antibiotics, and their results are applicable to slow-growing cells (their cells had a measured specific growth rate of 0.02/h prior to antibiotic addition), but they were not studying persister cells, and their conclusion that persister cells are metabolically active is not valid. Since their cell population was growing, they found the expected result that the cells were metabolically active. Also, since the cells were nutritionally stressed, they found the expected result that the RpoS-mediated stress response was important for the antibiotic tolerance.

In a similar manner, using biofilms generated for only 24 h, the Beloin group also rediscovered the importance of the RpoS-mediated stress response in nutritionally stressed cells that have antibiotic tolerance (25). In addition, they concluded that toxin/antitoxin systems were not involved in the antibiotic tolerance and that the stringent response (i.e., ppGpp) played less of a role than the stress response. Critically, their survival data for their biofilm population showed a dependence on antibiotic concentration, which clearly indicates that they were not studying persister cells (1); hence, their conclusions about the importance of RpoS versus the stringent response and of toxin/antitoxin systems are not valid for persister cells.

As an illustration of another important reason for differentiating persister cells from tolerant cells (26), the Balaban group has shown that tolerant cells can arise from cyclic antibiotic treatments that result in mutations that affect the duration of the lag phase (27). Persister cells, of course, tolerate antibiotics without undergoing mutation.

In conclusion, to distinguish between tolerant and persister cells and to avoid attributing traits of growing tolerant cells to dormant persister cells, groups should utilize the following techniques. (i) They should measure the number of putative tolerant cells (i.e., those cells that grow slowly prior to antibiotic treatment and then decrease slowly in the presence of the antibiotic) over a period of time; the number of persister cells should not increase prior to antibiotic treatment and should basically not change rapidly in the presence of the antibiotic, whereas the population of tolerant cells will increase prior to antibiotic addition and continue to decrease in the presence of the antibiotic (1). (ii) They should measure the number of putative tolerant cells as a function of antibiotic concentration above the MIC; the number of persister cells should not be a function of the antibiotic concentration as it is increased above the MIC, whereas the number of tolerant cells will decrease (1). (iii) They should ensure that the number of putative persister cells is not increasing; the number of persister cells will not increase in cell number in the absence of antibiotics and nutrients (but growing cells will increase in cell number). (iv) They should ensure that at no point is the persister population in contact with a medium that contains nutrients, even if only briefly (i.e., wash cells with nutrient-free buffer where required). In our lab, the surest way that we have found to produce high numbers of *E. coli* persister cells is to produce a toxin from a plasmid (28) or to pretreat the cells with compounds that reduce protein production (8). If the number of tolerant cells is increasing prior to antibiotic treatment, the authors will rediscover the well-known attributes of growing cells that are having their metabolism reduced; for example, they will discover that the growth/tolerance of the cells is dependent on stress sigma factors like RpoS. Therefore, the insights generated by the study of growing, tolerant cells are indeed worthwhile for the benefit of understanding nutritionally stressed cells; however, these findings should not be attributed to persister cells. Additionally, the terms persister and tolerant should not be used interchangeably, and authors should strive to indicate more clearly what type of research they are conducting. Using this rubric, i.e., distinguishing which cell population is being studied, many of the confusing aspects of persistence research, such as whether the cells have active metabolism, become clear: tolerant cells have some aspects of active metabolism, whereas persister cells, as originally determined, are dormant.
